# Use of C-Arm Cone Beam CT During Hepatic Radioembolization: Protocol Optimization for Extrahepatic Shunting and Parenchymal Enhancement

**DOI:** 10.1007/s00270-015-1146-8

**Published:** 2015-06-12

**Authors:** Andor F. van den Hoven, Jip F. Prince, Bart de Keizer, Evert-Jan P. A. Vonken, Rutger C. G. Bruijnen, Helena M. Verkooijen, Marnix G. E. H. Lam, Maurice A. A. J. van den Bosch

**Affiliations:** Department of Radiology and Nuclear Medicine, University Medical Center Utrecht, Room E.01.132, Heidelberglaan 100, 3584 CX Utrecht, The Netherlands

**Keywords:** C-arm CT, Cone beam CT, Radioembolization, IDEAL, Protocol optimization

## Abstract

**Purpose:**

To optimize a C-arm computed tomography (CT) protocol for radioembolization (RE), specifically for extrahepatic shunting and parenchymal enhancement.

**Materials and Methods:**

A prospective development study was performed per IDEAL recommendations. A literature-based protocol was applied in patients with unresectable and chemorefractory liver malignancies undergoing an angiography before radioembolization. Contrast and scan settings were adjusted stepwise and repeatedly reviewed in a consensus meeting. Afterwards, two independent raters analyzed all scans. A third rater evaluated the SPECT/CT scans as a reference standard for extrahepatic shunting and lack of target segment perfusion.

**Results:**

Fifty scans were obtained in 29 procedures. The first protocol, using a 6 s delay and 10 s scan, showed insufficient parenchymal enhancement. In the second protocol, the delay was determined by timing parenchymal enhancement on DSA power injection (median 8 s, range 4–10 s): enhancement improved, but breathing artifacts increased (from 0 to 27 %). Since the third protocol with a 5 s scan decremented subjective image quality, the second protocol was deemed optimal. Median CNR (range) was 1.7 (0.6–3.2), 2.2 (−1.4–4.0), and 2.1 (−0.3–3.0) for protocol 1, 2, and 3 (*p* = 0.80). Delineation of perfused segments was possible in 57, 73, and 44 % of scans (*p* = 0.13). In all C-arm CTs combined, the negative predictive value was 95 % for extrahepatic shunting and 83 % for lack of target segment perfusion.

**Conclusion:**

An optimized C-arm CT protocol was developed that can be used to detect extrahepatic shunts and non-perfusion of target segments during RE.

## Introduction

C-arm cone beam computed tomography (CT) can be used to acquire, reconstruct, and display high-resolution 3D images of selective contrast-enhanced vessels and the surrounding soft-tissue. Hence, it can provide valuable information on vascular anatomy and tissue perfusion during intra-arterial liver-directed treatments, such as trans-arterial chemoembolization (TACE) or radioembolization. However, the specific purpose for C-arm CT imaging may differ from treatment to treatment.

During TACE, C-arm CT is performed to identify the lesion of interest and all tumor-feeding arteries, to plan and navigate to the intended injection position, and to confirm adequacy of the injection position by evaluating contrast enhancement of the targeted tumor [1–4]. In contrast, during radioembolization, C-arm CT is used to map the hepatic arterial anatomy, to identify extrahepatic branches, and to rule out extrahepatic shunting or non-perfusion of a target volume [5, 6]. The latter allows for additional measures to be taken before the administration of technetium-99 m-labeled macro-albumin aggregates (^99m^Tc-MAA) and acquisition of single-photon emission computed tomography (SPECT). Since extrahepatic deposition of ^99m^Tc-MAA is reported in 10–20 % of patients after a workup solely based on digital subtraction angiography (DSA) [7, 8], C-arm CT could significantly reduce the number of angiography procedures that need to be repeated.

As these imaging purposes differ, they require other acquisition protocols, with appropriate timing of the contrast injection and scan delay. Thus far, an optimal acquisition protocol that enables the use of a single-run C-arm CT for radioembolization purposes has yet to be established. Furthermore, even though C-arm CT is increasingly performed, evidence for its added diagnostic value in radioembolization is still scarce. Thus, a clear need exists for the development and validation of a C-arm CT protocol for radioembolization.

The Innovation, Development, Exploration, Assessment, Long-term Study (IDEAL) recommendations describe how to perform a study with these aims [9]. These guidelines were initially formulated to provide a framework for a responsible stepwise evaluation of surgical innovations, but they also apply to complex interventions in the field of interventional radiology [10]. The first stage (‘Stage 1: Idea’) of evaluation is to perform a proof of concept study for a novel idea in a few selected patients. The next stage (‘Stage 2a: Development’) is the early development stage, in which prospective development studies should be performed in 10–100 patients to determine which technique has the best chance for procedural success, treatment efficacy, and safety. It is a crucial stage that differs the most from the pharmacological evaluations in the phase I–IV trial paradigm. “IDEAL supports prospective rather than retrospective studies at this stage, with sequential reporting of all cases and outcomes without omissions, and with clear explanations of when and how technique, design, or indications were changed” [11]. If these studies provide convincing evidence for safety and short-term benefits, the innovation enters the exploration stage (‘Stage 2b: Exploration’). The goal of this stage is to learn as much as possible about the safety and benefits of the procedure as patients and operators vary. Large, prospective, observational studies with registry data collection are particularly suited for this purpose. The last two stages (‘Stage 3: Assessment’ and ‘Stage 4: Long term study’) of evaluation are quite similar to pharmacological phase III and IV trials. Large (multicenter) randomized controlled trials are indispensable to assess comparative effectiveness of the innovation versus the current standard of care, and surveillance studies, preferably integrated in national patient registries, are needed to assess long-term safety and effectiveness outcomes [12].

The use of C-arm CT during radioembolization is still in ‘Stage 2A: Development.’ Accordingly, the purpose of this study was to develop a C-arm CT protocol optimized for the detection of extrahepatic shunting and non-perfusion of a target volume during radioembolization.

## Materials and Methods

### Study Design

A prospective development study was performed in accordance with phase 2A of the IDEAL recommendations, consisting of two parts: Part (1) prospective, stepwise, optimization of our C-arm CT protocol in clinical practice, and Part (2) blinded analysis of C-arm CT image quality and its diagnostic value.

C-arm CTs were already part of clinical practice during radioembolization procedures in our center, but the added value was limited to vascular mapping only, and the scan protocol was dependent on the operator. With the aim to reduce the number of repeat procedures for extrahepatic shunting or missed target segments, the protocol was optimized and then modified based on clinical experience. The medical ethics committee of the University Medical Center Utrecht waived the need for informed consent for reviewing imaging data in patients undergoing radioembolization in our center.

Reporting was done in agreement with the Strengthening the Reporting of Observational Studies in Epidemiology (STROBE) and the research reporting standards for radioembolization [13, 14].

### Study Population

All patients undergoing a pretreatment angiography during workup for radioembolization were eligible for C-arm CT acquisition, and thus for participation in our study. These patients had unresectable and chemorefractory liver malignancies (either primary tumors or metastasized), liver-dominant disease, a life expectancy exceeding three months, WHO performance status >2, hepatic tumor load ≤70 % of the liver volume, and unimpaired hepatic, renal, and hematological functions. Mirroring clinical practice, operators could refrain from acquiring a C-arm CT, if they deemed the additional contrast load too high in patients with previous allergic reactions, or when C-arm CT was considered of no added value in a particular patient (for example, in an ultra-selective injection position during segmental treatment of a single tumor).

### Technique, Equipment, and Scan Settings

The workup for radioembolization was performed following current standards of practice [15]. At baseline, patients received a ^18^F-FDG-PET scan combined with a multiphasic liver CT to (1) rule out contra-indications such as celiac axis stenosis, main portal vein thrombosis, or dominant extrahepatic disease, (2) localize the liver tumors, (3) assess the individual hepatic arterial anatomy, and (4) determine a patient-based treatment strategy [16]. During the pretreatment angiography, the hepatic arterial vasculature was selectively catheterized by femoral approach with a standard 5F guiding catheter (Celiac, Cobra or Simmons shape), 2.7F Progreat microcatheter (Terumo, Leuven, Belgium), and a 0.014-inch Transend guide wire (Boston Scientific, Natick, MA, USA). The celiac axis, common/proper hepatic artery, and left/middle/right hepatic arteries were selectively catheterized in all patients. The left gastric artery and superior mesenteric artery were only selectively catheterized when an aberrant hepatic artery was demonstrated on the pretreatment CT. Power injection DSA was used to search for potential sources of extrahepatic shunting. Coil embolization of extrahepatic branches was restricted to cases in which it was absolutely necessary to avoid extrahepatic shunting. Eventually, the microcatheter was positioned in the target vessel(s), and a C-arm CT acquisition was performed. A non-sequential, whole-liver approach with two or more selective injection positions was used in patients with bilobar disease. A lobar/segmental approach was used in patients with tumors confined to a single lobe or segment.

An Allura Xper FD20 (Philips, Best, The Netherlands) system, equipped with the XperCT and EmboGuide options, was used for the C-arm CT acquisitions. The abdomen fast high dose (HD) or abdomen fast low-dose (LD) settings were used. Depending on the setting, 312 (LD) or 624 (HD) images were acquired in a scan time of 5 (LD) or 10 (HD) seconds during a 240° rotation (Table 1).

**Table 1:** Differences between scan settings

Parameters	Abdomen fast high dose	Abdomen fast low dose
Rotation time	10.4 s	5.2 s
Number of images	624	312
Maximum rotation speed	20° per second	41° per second

Delay was defined as the time period between start of the contrast injection and start of the scan. Acquisition time was defined as the sum of the delay and scan time. Contrast agent (iodixanol 270 mg/ml, Visipaque 270; GE Healthcare) was diluted 1:1 with 0.9 % NaCl solution to reduce beam hardening artifacts and to limit the contrast burden. The injection of contrast agent was continued during the entire acquisition time in order to obtain images with contrast enhancement of the vascular tree and liver parenchyma. Injection rates were similar to those typically used in the common, proper, left, and right hepatic artery during power injector DSA.

The C-arm CT images were reviewed in the angio suite, and additional measures were taken if deemed necessary. Consequently, a total of 150 MBq ^99m^Tc-MAA were administered, and SPECT/CT images were acquired on a Symbia T16 scanner (Siemens Healthcare, Erlangen, Germany).

### Part 1: Protocol Optimization in Clinical Practice

Based on a literature review by two authors (AvdH, JP), a first protocol was defined. Starting from October 2013, all C-arm CTs were performed using this protocol.

After a predefined number of 5 patients, image quality and usefulness were subjectively assessed, and the need to alter one of the acquisition parameters was discussed in a consensus meeting between three investigators (AvdH, JP, EJV). This process was repeated until a protocol was found with sufficient image quality, allowing for both visualization of the arterial tree and detection of missed target segments and extrahepatic shunting. After optimization, further C-arm CTs were performed with that particular protocol, to expand the study population.

### Part 2: Retrospective Analysis of Image Quality and Diagnostic Value

Consequently, a retrospective analysis of image quality and diagnostic value was performed. For this purpose, scans were anonymized and randomized. For evaluation, Osirix (v.5.8 32-bit for MacOS X) was used. Reconstructions were made in the axial plane, using a window level of 60 HU and window width of 350 HU, with maximum intensity projections of 5 mm. Two raters (AvdH, JP) independently determined the vessel from which contrast was injected, the ability to discriminate between the perfused and non-perfused liver territory (categorized as “Yes”, “Partially”, and “No”), the presence of breathing artifacts, whether the field of view (FOV) contained the whole liver, the presence of extrahepatic shunting and the culprit vessel, and the presence of a non-perfused liver segment and if so, which one(s). Extrahepatic shunting was defined as a sharply defined area of contrast enhancement in the gastric wall, pancreas, duodenum, or bowel (excluding the gallbladder wall as an extrahepatic location). Discrepancies between the two raters were resolved during a subsequent consensus meeting.

For quantitative analyses, both raters drew six volumes of interest (VOIs), three in the perfused liver lobe and three in the non-perfused. Each VOI was placed in an area of the liver that was representative for the enhancement of the liver lobe, with a size between 1 and 10 cm^2^ to allow for proper estimation of the signal standard deviation (SD). Mean Hounsfield units (HU) and SD of the signal in the VOI were noted. The three VOIs were combined by averaging the three mean HU values. The SDs were combined using Eq. 1.

1 $$ {\text{SD}}_{\text{Combined}} = \sqrt {{\raise0.7ex\hbox{$1$} \!\mathord{\left/ {\vphantom {1 3}}\right.\kern-0pt} \!\lower0.7ex\hbox{$3$}}\,\,\, \times \,\,\left( {{\text{SD}}_{1}^{2} + {\text{SD}}_{2}^{2} + {\text{SD}}_{3}^{2} } \right)}. $$

For calculation of the signal to noise ratio (SNR), the following formula was used [17]:

2 $$ {\text{SNR}}\, = \,\frac{{{\text{Mean}} ({\text{HU}})}}{{{\text{SD}}\,\, ( {\text{HU)}}}}. $$

The contrast to noise ratio (CNR) was calculated to demonstrate differences between the perfused and non-perfused liver territories relative to the background noise in the liver parenchyma, by using the following formula [18]:

$$ {\text{CNR}} = \,\frac{{{\text{Mean}}_{\text{perfused}} {\text{ - Mean}}_{\text{non - perfused}} }}{{\sqrt {{\raise0.7ex\hbox{$ 1$} \!\mathord{\left/ {\vphantom { 12}}\right.\kern-0pt} \!\lower0.7ex\hbox{$ 2$}} \, \times \, \left( {SD_{\text{perfused}}^{ 2} { + }SD_{\text{non - perfused}}^{ 2} } \right)} }}. $$

The average of both raters was used as CNR to test for differences between the three protocols. All image quality analyses were performed on a scan basis.

SPECT/CT images were retrospectively reviewed by a nuclear medicine physician (BdK) with experience in the field of radioembolization (indication, patient management, administration, and evaluation). He was blinded for the outcome of the two raters evaluating the C-arm CT. The rater was asked to evaluate the presence of extrahepatic deposition in the gastric wall, pancreas, duodenum or bowel, and total lack of ^99m^Tc-MAA activity in one of the liver segments. This served as reference test to evaluate the diagnostic accuracy of C-arm CT. Negative predictive values (NPV) were determined for extrahepatic shunting and non-perfusion of a target volume. The diagnostic value analyses were performed on a procedure basis. For a procedure to be evaluated, C-arm CTs had to be acquired in all catheter positions in which ^99m^Tc-MAA was injected.

### Statistics

Differences in CNR between the three protocols were tested using an ANOVA. A Fisher–Freeman–Halton exact test was used to compare the subjective score for discriminating ability between protocols. A *p* value < 0.05 was considered statistically significant. All statistical analyses were performed with R version 3.0.1 for Windows.

## Results

### Study Population

**Fig. 1:**
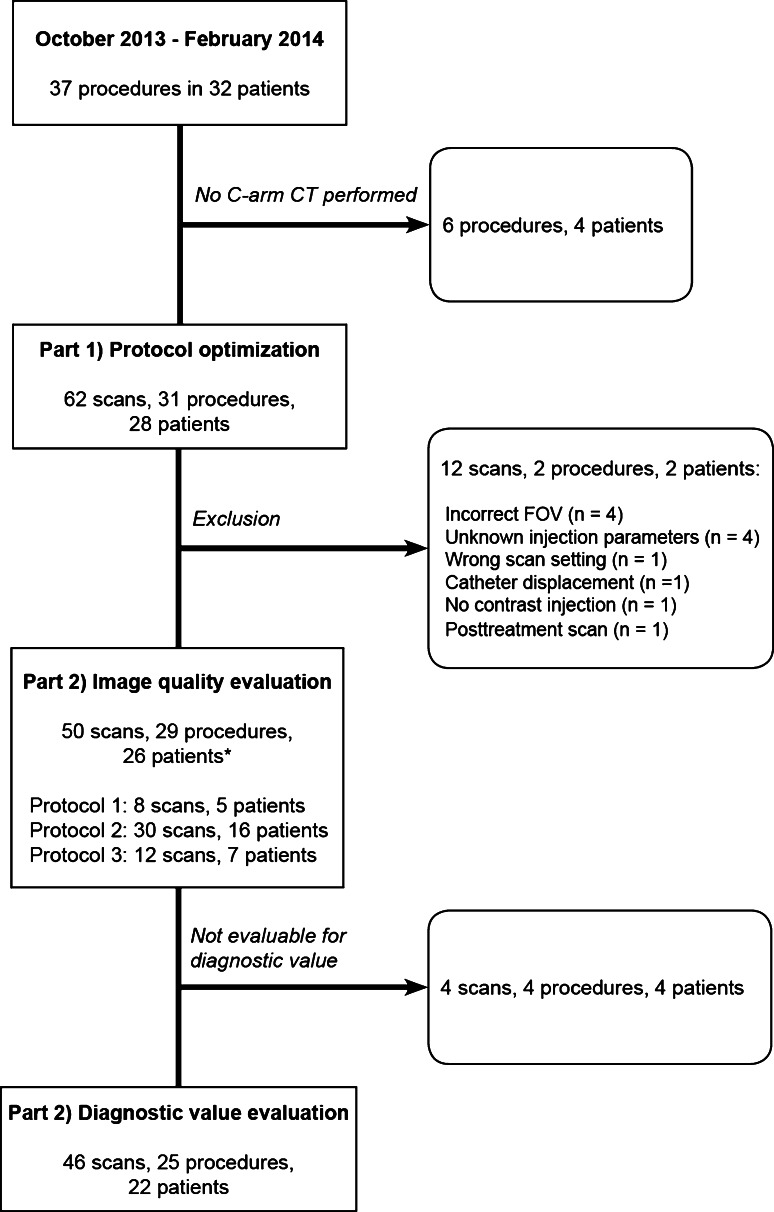
Detailed flowchart of the selection process showing the number of scans, procedures, and patients. *Asterisk* indicates that the number of unique patients is 26. Two patients were scanned with more than one protocol

A flowchart of this study is displayed in Fig. 1. From October 2013 until February 2014, we performed 37 pretreatment angiographies in 32 patients in our institute. During 31 angiography procedures, a total of 62 C-arm CTs were obtained in 28 different patients. The majority of C-arm CTs were performed during the first pretreatment angiography (*n* = 26), four were performed during repeat procedures, and 1 during the second pretreatment angiography for the treatment of a different liver lobe (*n* = 1).

Twelve C-arm CTs had to be excluded for the following reasons: the FOV was incorrect (*n* = 4 scans), the injection parameters were unknown (*n* = 4), the catheter was displaced during contrast injection (*n* = 1), the wrong acquisition settings were used (*n* = 1), the scan was made after treatment (*n* = 1), and no contrast was injected (*n* = 1). For two procedures, these scans were the only C-arm CTs available; these two patients were excluded, leaving a total of 50 scans acquired during 29 procedures in 26 patients for analysis.

The patients included in this study had a median age of 64 years (range 45–80 years), and 16/26 (62 %) were male. They were treated for primary or metastatic liver tumors, with the following primary tumor types: colorectal cancer (*n* = 10, 37 %), hepatocellular carcinoma (*n* = 6, 23 %), cholangiocarcinoma (*n* = 2, 8 %), breast cancer (*n* = 2, 8 %), and others (*n* = 6, 24 %). Median tumor burden was 14 % (range 0–66 %), WHO performance score was 0 in 18 patients (69 %), 1 in 3 patients (12 %), 2 in 1 patient (4 %), and not reported in 4 patients (15 %). Child Pugh score was A5 in the majority of patients (*n* = 24, 92 %), A6 in one patient (4 %), and not reported in another patient (4 %).

### Part 1: Protocol Optimization in Clinical Practice

**Fig. 2:**
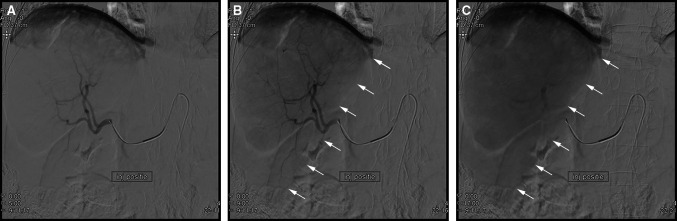
Example of how the C-arm CT scan delay was determined on power injection DSA in the right hepatic artery. **A** Start of the DSA run. Only vascular contrast enhancement is visible. **B** Midway the DSA run. Parenchymal contrast enhancement of the right liver lobe (*white arrows* indicate the border of the right liver lobe) is starting to show. **C** At the end of the DSA run, maximal parenchymal contrast enhancement is reached. The time between the first and last run is used as delay for the C-arm CT scan

For the first protocol, a fixed delay of 6 s was chosen based on a literature review, combined with the high-dose scan setting of 10 s. After the first protocol was applied during 5 procedures (8 C-arm CTs, 5 unique patients), the degree of parenchymal contrast enhancement was deemed insufficient. To improve the parenchymal enhancement, it was decided to use a variable delay. This delay was determined by assessing the time to parenchymal enhancement on power injection DSA, using an identical injection rate and catheter position (Fig. 2). This method adjusts for differences between liver lobes and between patients.

For the second protocol, a variable delay (median 8 s, range 3–10 s) and 10-s high-dose scan setting were used. This protocol was used during 10 procedures (17 scans in 9 patients). Parenchymal contrast enhancement had improved substantially in comparison with the first protocol. However, the relatively long scan time was associated with breathing artifacts (from 0 to 27 %).

In the third protocol, used during 7 procedures (12 scans in 7 patients), a 5 s low-dose scan setting was applied to reduce breathing artifacts, in combination with a variable delay (median 8 s, range 4–10 s). It did show considerably less breathing artifacts, and parenchymal contrast enhancement was acceptable. Still, this protocol was not favored over the second protocol, because the low-dose scan settings had led to deterioration of the overall image quality.

The second protocol was subsequently used in another 7 procedures (13 scans in 7 patients), bringing it to a total use in 17 procedures (30 scans in 16 patients).

### Part 2: Retrospective Analysis of Image Quality and Diagnostic Value

For image quality analysis, eight of these scans were obtained with protocol 1, 30 scans with protocol 2, and 12 scans with protocol 3.

The results of the image quality analysis are summarized in Table 2. The median CNR (range) for discrimination between the perfused and non-perfused liver territories was 1.7 (0.6–3.2) for protocol 1, 2.2 (−1.4 to 4.0) for protocol 2, and 2.1 (−0.3–3.0) for protocol 3 (*p* = 0.80). The subjective score for discriminating ability was Yes—Partially—No, in 57 %—29 %—14 % of evaluable scans for protocol 1, 73 %—27 %—0 % of scans for protocol 2, and 44 %—33 %—22 % of scans for protocol 3 (*p* = 0.13). Nine scans (2× protocol 1, 4× protocol 2, 3× protocol 3) could not be evaluated for the discriminating ability due to the absence of a non-perfused territory. Breathing artifacts were reported in none of the scans for protocol 1, 8/30 scans (27 %) for protocol 2, and 1/12 (8 %) scans for protocol 3.

**Table 2:** Summary of protocols and outcomes

Protocol				Number of Scans	CNR, median (range)	Subjective discriminating ability			
#	Delay	Scan time (s)	Scan setting			Yes, *n* scans (%)	Partially, *n* scans (%)	No, *n* scans (%)	Not evaluable, *n* scans
1	6 s	10	Fast HD	8	1.7 (0.6–3.2)	4 (57 %)	2 (29 %)	1 (14 %)	1
2	Variable^a^	10	Fast HD	30	2.2 (−1.4–4.0)	19 (73 %)	7 (27 %)	0 (0 %)	4
3	Variable^a^	5	Fast LD	12	2.1 (−0.3–3.0)	4 (44 %)	3 (33 %)	2 (22 %)	3

The protocol settings, number of scans per protocol, objective (CNR), and subjective ability to discriminate between perfused and non-perfused liver territories are displayed in Table 1

*CNR* contrast to noise ratio, *HD* high dose, *LD* low dose

^a^Estimated by contrast-enhanced DSA series where time between infusion and liver parenchyma enhancement is used as the delay time for C-arm CT

For diagnostic accuracy analysis, 25/29 (86 %) procedures were evaluable: a C-arm CT was not obtained in all injection positions in 2 procedures, a dissection hampered SPECT/CT acquisition in 1 procedure, and C-arm CTs were not assessable due to breathing artifacts in 1 procedure.

**Fig. 3:**
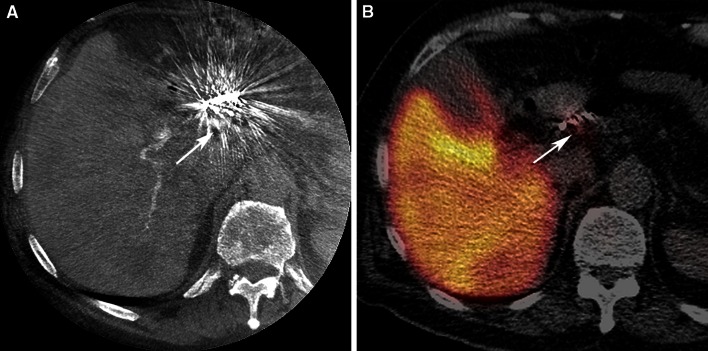
**A** Extrahepatic deposition of ^99m^Tc-MAA activity in the region of the coil embolized right gastric artery on a fusion SPECT/CT image (*white arrow*). **B** On C-arm CT imaging, no extrahepatic shunting was noted, due to the extensive coil-related beam hardening artifacts (*white arrow*)

**Fig. 4:**
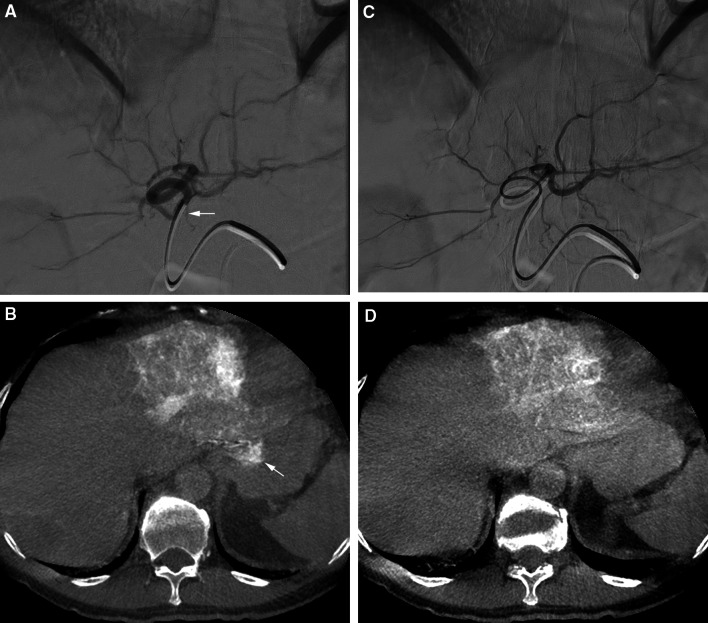
**A** DSA from the LHA. **B** C-arm CT performed from the LHA shows extrahepatic shunting in the gastric wall (*white arrow*). The small extrahepatic branch indicated by the *white arrow* in (**A**) was the culprit vessel. **C** The catheter was positioned more distal in the LHA. **D** C-arm CT performed from the new injection position did not show extrahepatic shunting anymore

In 21/25 procedures (84 %, Table 3), the retrospective C-arm CT analysis revealed no extrahepatic shunting. In one of the 21 procedures, SPECT/CT analysis demonstrated extrahepatic deposition of ^99m^Tc-MAA. This extrahepatic deposition occurred near the implanted coils in the right gastric artery and could not be detected on the C-arm CT (Fig. 3). The negative predictive value for extrahepatic shunting was 95 %. It should be noted that during 3 procedures (14 %), extrahepatic shunting had already been observed on C-arm CT in clinical practice. In two of these patients, an extrahepatic branch (pancreatic/duodenal branch from the RHA, collateral between cystic artery and the gastroduodenal artery) was successfully coil embolized, before the administration of ^99m^Tc-MAA. In the other patient, the catheter was positioned distal to the extrahepatic branch (gastric branch originating from the LHA). Success was confirmed by a repeated C-arm CT scan without extrahepatic shunting (see Fig. 4 for an example).

**Table 3:** Diagnostic outcomes—gastrointestinal shunting

Gastrointestinal shunting		^99m^Tc-MAA SPECT/CT		
		Present	Absent	Total
C-arm CT	Present	3	1	4
	Absent	1	20	21
	Total	4	21	25
	Negative predictive value			95.2 %

Two by two table displaying the presence and absence of gastrointestinal shunting on C-arm CT (experimental test) and ^99m^Tc-MAA SPECT/CT (reference standard). The numbers represent the number of procedures

**Fig. 5:**
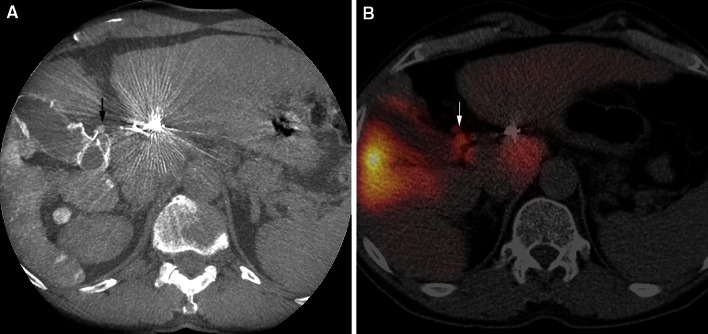
Comparison of C-arm CT and SPECT/CT in a patient with extrahepatic shunting. **A** C-arm CT shows extrahepatic shunting in the duodenal region (*black arrow*), caused by a collateral branch from the cystic artery. **B** Corresponding extrahepatic ^99m^Tc-MAA activity in the duodenal region on SPECT/CT (*white arrow*)

In our retrospective evaluation, C-arm CT showed extrahepatic shunting in 4/25 procedures (16 %), located in the duodenal region (*n* = 3) or stomach wall (*n* = 1). The SPECT/CT analysis confirmed extrahepatic deposition in 3 of these procedures (Fig. 5).

**Fig. 6:**
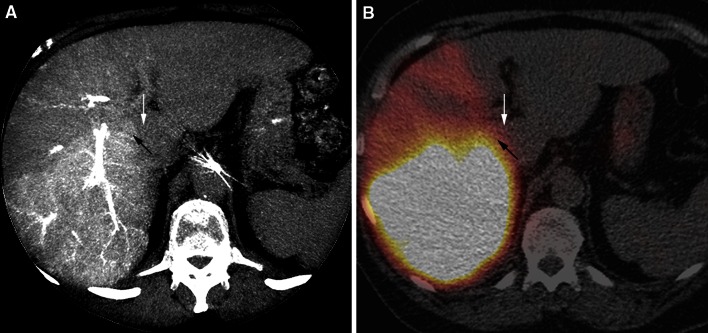
Comparison of C-arm CT **A** and SPECT/CT **B** in a patient with right lobar infusion of ^99m^Tc-MAA. The right part of segment 1 is perfused (*black arrow*), but the left part is non-perfused (*white arrow*). Note the free pertechnetate in the stomach on SPECT/CT (**B**)

Out of the 25 procedures that were assessable for the perfusion of target liver territories, 7 (28 %) showed one or more unperfused target segments in the C-arm CT analysis (Table 4). Five out of those 7 procedures also showed a lack of perfusion on ^99m^Tc-MAA (Fig. 6). In the two remaining patients, segments I and IV showed no contrast enhancement on C-arm CT, but ^99m^Tc-MAA activity was visible on SPECT/CT. Both patients had markedly hypervascular tumors with a heterogeneous contrast and ^99m^Tc-MAA activity distribution. Furthermore, in 3/18 procedures with adequate perfusion on C-arm CT, lack of ^99m^Tc-MAA activity was found on SPECT/CT (in segments I-IVb, segment VII, and segments I-V + VIII) in the retrospective analysis. The negative predictive value for non-perfusion was 83 %.

**Table 4:** Diagnostic outcomes—non-perfused target volumes

Non-perfused target volume		^99m^Tc-MAA SPECT/CT		
		Present	Absent	Total
C-arm CT	Present	5	2	7
	Absent	3	15	18
	Total	8	17	25
	Negative predictive value			83.3 %

Two by two table displaying the presence and absence of non-perfused target volumes on C-arm CT (experimental test) and ^99m^Tc-MAA SPECT/CT (reference standard). The numbers represent the number of procedures

## Discussion

In this study, an acquisition protocol for C-arm CT imaging has been developed that meets specific needs during radioembolization procedures.

We have shown that a continuous infusion of contrast agent, a variable scan delay based on the time to parenchymal enhancement on DSA, and a 10 s high-dose scan setting resulted in images that contain both contrast enhancement of the arterial tree and liver parenchyma show gastrointestinal shunting, and provide sufficient contrast between perfused and non-perfused liver territories, all in a single C-arm CT run. It is expected that optimization of these acquisition parameters increases the detection rate of angiographic failures, providing an opportunity to take additional measures and prevent unnecessary repeat procedures.

In our series, the NPV for extrahepatic shunting and non-perfusion were 95 and 83 % respectively, which is in line with the results of two previous studies. In 2009, Louie et al. performed a study in 42 patients who underwent radioembolization for primary and metastatic liver tumors. In a total of 22/42 patients (52 %), extrahepatic shunting or incomplete tumor perfusion on C-arm CT affected the treatment plan. In the majority (14/22 patients), these findings were not detected on DSA. Extrahepatic shunting was demonstrated on C-arm CT in 8 patients (19 %), and only in 1 on SPECT/CT. According to the authors, this incongruence can be explained by the limited spatial resolution of SPECT/CT. Interestingly, 1 patient with extrahepatic shunting on C-arm CT developed a gastric ulcer upon follow-up, as a complication of extrahepatic yttrium-90 microsphere deposition [5].

Later, Heusner et al. assessed the accuracy of C-arm CT for the detection of extrahepatic shunting before RE in 30 patients with primary and metastatic liver tumors in a similar type of study. Using ^99m^Tc-MAA SPECT/CT as reference standard, they found a negative predictive value of 96 %, and C-arm CT detected extrahepatic shunting that was not visible on DSA in 10 % of their patients [6].

Other studies reported that arterial and parenchymal enhancement images can also be acquired in two separate C-arm CT scans using two different delay times, or by means of a customized dual-phase C-arm CT setting that allows back-to-back acquisitions of two scans with a single contrast injection [3, 19]. In our opinion, it is easier to continue the contrast infusion during the entire acquisition time (delay + scan time). This also provides the benefit that potential extrahepatic shunting and the culprit vessel can be identified in the same image.

Now the technical aspects have been refined and the feasibility of C-arm CT as a diagnostic tool is demonstrated; there is a window of opportunity to rigorously test its diagnostic value in accordance with ‘Stage 2B: Exploration’ and ‘Stage 3: Assessment’ of the IDEAL recommendations. C-arm CT is not likely to replace the infusion of ^99m^Tc-MAA, for the latter is also used for the evaluation of lung shunting and dosimetric evaluation. Nevertheless, C-arm CT may come to play an important role in evaluating the pretreatment angiographies, since it allows for timely intervention to prevent repeat angiographies. Furthermore, C-arm CT is, as an adjunct to a multiphasic pretreatment CT, indispensable in the development of a single-day treatment algorithm for radioembolization [20].

The current study suffers from a relatively small sample size, as is common in the developmental phase of a new technique. To prevent bias, all consecutive cases were described and their reasons for inclusion/exclusion were mentioned. Also, there was no predefined plan for the modifications to the scan protocol, it was adjusted by the needs identified by the consensus meeting. Furthermore, there is no clear end point for an optimal scan protocol, so other studies may improve on this proposal. For our diagnostic accuracy evaluation, it was not possible to assess the false positive rate for C-arm CT, since additional measures were taken when extrahepatic shunting was detected during the preparatory angiography. Besides, the operator was allowed to refrain from using C-arm CT. In theory, this may introduce selection bias. However, in most cases, there was a reason to refrain from C-arm CT (e.g., contrast allergy). Finally, ^99m^Tc-MAA SPECT/CT was used as a reference standard, but this modality also has limitations. Its limited spatial resolution and room for registration errors between SPECT and CT volumes can make the detection of extrahepatic deposition a challenging task. Furthermore, ^99m^Tc-MAA SPECT/CT is an imperfect predictor for the posttreatment yttrium-90 microsphere distribution. Therefore, future investigations should determine the definite role of C-arm CT and ^99m^Tc-MAA SPECT/CT in the workup for radioembolization.

In conclusion, we have developed an optimized C-arm CT protocol that can be used to detect extrahepatic shunts and non-perfusion of target segments during RE. Its use is currently in the developmental phase, and needs to be further evaluated in the near future.
